# Effects oflysophospholipids supplementation on production performance, serum biochemistry, rumen fermentation in lactating dairy cows

**DOI:** 10.3389/fvets.2026.1912675

**Published:** 2026-09-17

**Authors:** Ruoran Tao, Tao Wang, Sijia Liu, Dian Wang, Zhiyuan Xu, Maocheng Jiang, Yinghao Huang, Caiyun Fan, Zhao Zhuo, Jianbo Cheng

**Affiliations:** 1 College of Animal Science and Technology, Anhui Agricultural University, Hefei, China; 2 National Center of Technology Innovation for Dairy, Hohhot, China; 3 Inner Mongolia Youran Dairy Group Limited, Hohhot, China

**Keywords:** Holstein cows, lysophospholipids, microorganisms, production performance, rumen fermentation, serum biochemistry

## Abstract

**Objective:**

This study was designed to evaluate the effects of supplementing dairy cow diets with lysophosphatidylcholine (LPL) on production performance, serum biochemical parameters, and rumen fermentation profiles.

**Methods:**

A total of 20 Holstein dairy cows in the early lactation stage with comparable body conditions (DIM = 73 ± 26 d; milk yield = 41 ± 10 kg; average parity = 2.6 ± 1.1) were selected and randomly assigned to two groups:the control group (CON), which received a basal diet only, and the experimental group (LPL), which was supplemented with 10 g/day of lysophosphatidylcholine (LPL) in addition to the basal diet. The experimental period lasted for 60 days. At the conclusion of the trial, samples were collected for the assessment of apparent digestibility, production performance, serum biochemical parameters, and rumen fermentation profiles.

**Results:**

The results demonstrated that LPL supplementation significantly increased milk yield, milk fat percentage, and milk protein concentration. It also improved the apparent digestibility of crude fat and enhanced rumen fermentation by increasing propionate and microbial crude protein levels while decreasing ammonia nitrogen concentration. Blood analysis revealed elevated levels of glucose and non-esterified fatty acids (NEFA), along with reduced concentrations of triglycerides (TG) and blood urea nitrogen (BUN). Although LPL had minimal impact on overall rumen microbial diversity, it altered the relative abundance of specific bacterial species.

**Conclusion:**

Dietary supplementation with 10 g/day LPL improved milk yield, milk fat percentage, milk protein percentage, and and apparent EE digestibility in lactating dairy cows. LPL also altered selected serum biochemical parameters related to glucose and lipid metabolism, while showing limited effects on overall rumen bacterial diversity and community structure.

## 1 Introduction

In dairy cows, fat serves as the primary energy reserve and is the nutrient with the highest energy density in feed (1), while also enhancing feed palatability (2). Furthermore, fat functions as a carrier for fat-soluble vitamins, facilitating their absorption, which underscores its importance in maintaining normal physiological functions. During early lactation, milk production increases rapidly, accompanied by heightened metabolic and nutritional demands. When cows are unable to consume sufficient feed to meet these energy requirements—particularly when the energy needed for milk production and maintenance exceeds intake—they experience negative energy balance (NEB). NEB triggers the mobilization of body energy reserves, primarily from adipose tissue, to fulfill the high energy demands during early lactation. Fat supplementation and dietary enrichment are therefore critical strategies for meeting energy needs during this phase. However, due to limitations in digestive capacity and economic constraints, the extent and scope offat inclusion indiets are restricted. However, excessive dietary fat supplementation may reduce DM intake and disturb ruminal fermentation or fiber digestion, depending on the amount, source and fatty acid profile of the far supplement (3–5). Therefore, improving dietary fat digestibility while avoiding excessive fat conclusion remains an important strategy in dairy cow nutrition. One effective approach involves incorporating emulsifiers into animal diets, among which lysophosphatidylcholine (LPL) has emerged as a promising candidate. LPL can act as a biosurfactant/emulsifier and has been reported to improve lipid emulsification, digestion, and nutrient utilization by increasing the contact between lipid substrates and digestive enzymes (6–8).

LPL is a monoglycerophosphocholine ester compound (9) structurally similar to common phospholipids, possessing both hydrophilic and lipophilic functional groups. Its distinguishing feature lies in the reduced number of hydrophobic groups, significantly enhancing its hydrophilicity and water dispersibility compared to conventional phospholipids. This property enables LPL to function more effectively as a feed additive, promoting fat digestion and absorption while enhancing feed nutritional value. Research has shown that supplementing lactating cow diets with varying doses of LPL significantly improves milk yield, milk composition, and nitrogen utilization, with a dry matter inclusion rate of 0. 075% proving more effective than 0. 05% (7). Additionally, LPL exhibits potential in modulating muscle lipid composition; its dietary inclusion reduces serum lipase concentration, possibly promoting intramuscular lipid deposition and improving meat quality (10). In terms of antioxidant activity, LPL demonstrates efficacy comparable to vitamin C in inhibiting lipid peroxidation (11), highlighting its significant application potential in dairy production as an effective nutritional additive capable of enhancing both productivity and animal health. Studies on monogastric animals have further supported the benefits of LPL. For instance, Xing et al. (12) reported that adding 0. 02%LPL and 5% lard to weaned piglet diets significantly improved digestibility: dry matter digestibility increased by 5.21%, fat digestibility by 10.6%, total energy digestibility by 2.74%, and protein digestibility by 7.21%. Jones et al. (6) found that LPL supplementation in piglet diets enhanced fat utilization by 3.46%. In poultry, LPL has been shown to improve the digestion and absorption of dry matter, crude fat, and protein in broilers, particularly when incorporated into low-energy diets, which enhances growth performance and supports intestinal health (13, 14). Zangeneh et al. (15) observed that supplementing broiler diets with 0. 1% LPL increased average daily weight gain by 4.6%, elevated bursa of Fabricius and thymus indices, and boosted antibody concentrations against sheep red blood cells, Newcastle disease virus, and infectious bronchitis virus in the blood, thereby promoting overall immunity. Additional studies have similarly indicated that LPL supplementation in broiler diets enhances body weight, antibody titers against Newcastle disease and infectious bursal disease, and improves feed conversion efficiency and carcass quality (16–18).

Although LPL has been evaluated in several animal species, it remains unclear in lactating dairy cows whether changes in production performance are primarily associated with improved dietary fat utilization and systemic metabolic responses or with alterations in rumen fermentation and microbial ecology. We hypothesized that supplementation with 10 g/d LPL would improve lactation performance and apparent EE digestibility and would be accompanied by changes in selected serum biochemical and rumen fermentation indices, while exerting limited effects on overall rumen bacterial diversity and community structure. Therefore, this study evaluated milk production and composition, apparent nutrient digestibility, serum biochemical indices, rumen fermentation characteristics, and rumen bacterial communities in lactating dairy cows.

## 2 Materials and methods

### 2.1 Animals, experimental design, and diets

This study was conducted at an ecologicall famm in Shandong Province, China, Twenty healthy lactating Holstein cows-characterized by‘ mean DIM of 73 ± 26 d, average daily milk yield of 41 ±10, kg and parity of 2.6 ± 1.1- were enrolled. Animals were assigned to either the CON group or the LPL group using a completely randomized design, with 10 cows per group. Baseline DIM, milk yield, and parity showed no statistically significant differences between groups (*P* > 0.05), confirming successful balance across key physiological parameters. The CON group received the Basal diet only, whereas the LPL group received the same basal diet supplemented with 10 g/d of a commercial lysophospholipid product [Bylow Hualian Chemical (Shanghai) Co., Ltd., Shanghai, China]. This product is enzymatically modified from soybean lecithin and contains primarily lysophosphatidylcholine, lysophosphatidylinositol, and lysophosphatidic acid as active components. The 10 g/d dosage was selected based on dose-response datal from prior *in vivo* studies in dairy cattle and aligned with the manufacturer's recommended inclusion level for nutritional supplement (7, 19). Table 1 presents the dietary composition and nutrient levels, which met the daily nutritional requirements of dairy cows as recommended by NRC (20). Throughout the experimental period, cows were housed in individual stalls distributed across the barn, with each treatment group assigned to a designated area within the facility. The animals were allowed unrestricted movement and had continuous access to clean drinking water. The total mixed ration (TMR) was provided at 110% of the cows' estimated intake requirements to ensure *ad libitum* feeding. Cows were fed three times daily at 05:00, 13:00, and 21:00. The health status of each cow was monitored on a daily basis, and regular communication with veterinarians was maintained throughout the study to ensure optimal animal health.

**Table 1:** TMR composition and nutritional components (dry matter basis).

Feed ingredients	Proportion, %	Nutrient composition^3^	Content, %
Corn silage	30.6	NEL^2^, Mcal/kg	1.72
Alfalfa hay	13.3	CP	16.83
Imported oats	5.5	Ether extract	2.54
Beer grains	5.5	NDF	36.
Compressed corn flakes	7.1	ADF	8,928.
Beet pulp	5.5	Ca	21
All-cotton meal	4.3	P	0.78
Soybean meal	12.2		0.47
Concentrate supplement^1^	16.0		
Total	100		

^1)^The concentrate supplement consisted mainly of corn grain, soybean meal, cottonseed meal, minerals, and vitamins. One kg premix contained the following: VA 400000 IU, VD60000 IU, Fe 400 mg, Cu 250 mg, Zn 3000 mg, Mn 350; 1 kg of concentrate supplement provided vitamin A 400,000 IU, vitamin D 60,000 IU, Fe 400 mg, Cu 250 mg, Zn 3,000 mg, Mn 350 mg, Se 15 mg, and Co 35 mg.

^2)^Estimated using the Natio nal Acade m ies of Sc ie nces, Engi nee r ing, and Medici ne (5) model based on DM intakes.

^3)^The nutrient levels are measured values.

### 2.2 Data collection and sampling procedure

During the experiment, cows were housed in a free-stall system. The total mixed ration (TMR) was prepared using a TMR mixer at designated times and locations each day and distributed to the cattle barn. Additives were administered separately to the experimental cattle, commencing at 12:00 daily and provided once per day. The required additive amounts for each group of cattle were prepped 1 day in advance according to their respective groupings and made ready for administration. Throughout the experiment, the cattle had *ad libitum* access to both feed and water. The leftover feed from the previous day was collected prior to morning feeding the following day, and its quantity was recorded to determine the actual feed intake of the previous day. The cattle barn was cleaned of manure once daily to maintain optimal hygiene conditions.

During the experiment, three TMR samples were collected daily and thoroughly mixed to analyze the feed composition. The samples were dried at 65 °C for 48 h until a constant weight was attained. Subsequently, the dried TMR samples were ground using a 1 mm sieve (KRT-34; Beijing Kunjie), and the dry matter (DM) content was determined according to AOAC method 950.15. Nitrogen content was measured using AOAC method 984.13 (21), and crude protein (CP) was calculated by multiplying the nitrogen content by 6.25. Neutral detergent fiber (NDF) and acid detergent fiber (ADF) contentswere analyzed following the procedure described by Van Soest et al. (22). Ash content was determined in accordance with AOAC method 942.05 (21).

On the 60th day, fecal grab samples were collected from these selected cows four times a day (at 3 a.m., 8 a.m., 1 p.m., and 6 p.m.). The samples from each cow were thoroughly mixed to form a simple composite sample, and about 200 grams were taken. One quarter of the weight of the feces was added with 10 % tartaric acid to fix nitrogen. These fecal grab samples were placed in a forced ventilation oven at 65 °C until completely dry, and then ground to pass through a 1 mm sieve (KRT - 34; Beijing Kunjie, China). The fecal DM, CP, and EE contents were analyzed according to AOAC International (21), whereas fecal NDF and ADF were analyzed following Van Soest et al. (22). AIA was used as an internal marker to determine apparent nutrient digestibility.

The total mixed ration (TMR), leftover feed, and fecal samples were analyzed according to the method of Van Keulen and Young (23). The calculation method of the apparent digestibility of nutrients is as follows:

1. Apparent digestibility of nutrients (%) = [1–(Ad x Nf) / (Af x Nd)] x 100.

Where Ad and Af represent the AIA content in feed and feces (g/ kg), respectively; Nf and Nd represent the content of the corresponding nutrient in feces and feed (g/ kg), respectively.

The daily milk yield of each cow was recorded, and the weekly average was calculated. Every week, 100 ml of milk samples were collected from each cow during each milking session. Milk samples collected in the morning and afternoon were stored at 4 °C and later combined with the evening samples from the same cow for analysis using a milk composition analyzer (BactoScanFCCombiFoss 6,000; Foss Electric, Hillerød, Denmark). The analyzer measured the content of milk fat, protein, lactose, total solids, urea nitrogen, and somatic cell count (SCC).

On the 60th day of the experimental period, eight cows were randomly selected from each group (the same 162 individuals from which fecal samples were collected), and blood was drawn from the caudal vein prior to morning feeding. Blood samples were collected into both biochemical tubes and sodium heparin anticoagulant tubes. The samples in the biochemical tubes were centrifuged at 1,370 ( × g) for 10 min, and the resulting serum was divided into four 1.5 ml aliquots and stored at −20 °C for subsequent analysis. The measured indicators Included total protein (TP), albumin (ALB), blood glucose (GLU), total cholesterol (TC), triglycerides (TG), alanine aminotransferase (ALT), aspartate aminotransferase (AST), high-density lipoprotein (HDL-C), low- density lipoprotein (LDL-C), non-esterified fatty acids (NEFA), insulin (INS), blood urea nitrogen (BUN), and β-hydroxybutyrate (BHBA). All biochemical indicators were analyzed using an automatic biochemical analyzer (Synchron CX5 Pro, USA) in accordance with the instructions provided by the corresponding detection kits.

On the 60th day of the experiment, rumen fluid was collected from the cows 2 h after morning feeding. Six cows were randomly selected from each of the control and experimental groups for sample collection. To prevent saliva contamination, the first 200 ml of rumen fluid was discarded, and then approximately 200 mL of fluid was retained. The pH value was immediately measured after collection. Ten milliliters of each sample was transferred to a centrifuge tube and stored at −20 °C for subsequent determination of the concentrations of volatile fatty acids (VFA), ammonia nitrogen (NH_3_ -N), and microbial crude protein (MCP).

Meanwhile, the filtered rumen fluid was aliquoted into cryotubes (two tubes per cow) and stored in liquid nitrogen for microbial community analysis. The concentration of VFA was determined by high-performance liquid chromatography (HPLC, Agilent 1,100, USA). Specifically, 20 ml of the rumen fluid sample was centrifuged at 1,006 ( × g) for 15 min. Then, 10 mL of the supernatant was transferred to a 20 ml centrifuge tube, followed by the addition of 2 ml of 20% zinc sulfate and 1 ml of 10% potassium ferricyanide. The mixture was homogenized using a vortex oscillator and subsequently centrifuged at 11,180 ( × g) at −4 °C for 15 min. The supernatant was then filtered through a 0.22 μm water system filter membrane before HPLC analysis. The chromatographic conditions were as follows: column, TC-C18(150 mm × 4.6 mm, 5 μm); column temperature, 30 °C; mobile phase, 0.01 mol/L sodium dihydrogen phosphate, methanol, and ultrapure water; injection volume, 3 μL; UV detector wavelength, 213 nm. The concentration of NH3-N was determined using the phenol-sodium hypochlorite method, while MCP was quantified using the Coomassie Brilliant Blue G250 method.

### 2.3 Rumen microbiota 16S rRNA sequencing and analysis

#### 2.3.1 DNA extraction and PCR amplification

One milliliter ofrumen fluid was thawed at 4 °C, and genomic DNA was extracted using the FastDNA^®^ Spin Kit for Soil (Megazyme, China) according to the manufacturer's instructions. The concentration and purity of the extracted DNA were assessed using a NanoDrop 2000 ultramicro spectrophotometer (Thermo Fisher Scientific, USA). The V3–V4 hypervariable region of the bacterial 16S rRNA gene was selected as the target fragment and amplified via PCR using the forward primer 338F 5′-ACTCCTACGGGAGGCAGCAG-3′) and reverse primer 806 R (5′–GGACTACHVGGGTWTCTAAT–3′) on an ABI GeneAmp^®^ 97 00 PCR system (ABI, USA). The PCR cycling conditions included initial denaturation at 95 °C for 3 min; 30 cycles of denaturation at 95 °C for 30 s, annealing at 55 °C for 30 s, and extension at 72 °C for 45 s; followed by a final extension at 72 °C for 10 min. PCR products were analyzed, purified, and quantified using 2% agarose gel electrophoresis and a DNA gel recovery purification kit (PCR Clean-Up Kit, China Yu Hua). The purified amplicons were further quantified using a Qubit 4.0 fluorometer (Thermo Fisher Scientific, USA).

#### 2.3.2 Analysis of sequencing data

Purified amplicons were pooled in equimolar amounts and paired-end sequenced on an Illumina Nextseq2000 platform (Illumina, San Diego, USA) according to the standard protocols by Majorbio Bio-Pharm Technology Co. Ltd. (Shanghai, China). The raw sequencing reads were deposited into the NCBI Sequence Read Archive (SRA) database (Accession Number: SRP1290675). Reviewer links for PRJNA1290675: https://www.ncbi.nlm.nih.gov/sra/?term=PRJNA1290675 The datasets used and/or analyzed during the current study are available from the corresponding author on reasonable request.

### 2.4 Statistical analyses

Statistical Analyses: Data were analyzed using SPSS 25.0 (IBM Corp., Armonk, NY, USA). For repeatedly measured variables, including milk yield and milk composition, data should be analyzed using a mixed model with treatment, time, and treatment × time interaction as fixed effects and cow as a random effect. Endpoint variables, including apparent digestibility, serum biochemical parameters, and rumen fermentation parameters, may be analyzed using an independent-samples *t*-test if normality and homogeneity of variance are satisfied; otherwise, appropriate non-parametric tests or transformations should be used. Data should be presented as means ± SEM, and significance should be declared at *P* < 0.05.

## 3 Results

### 3.1 Milk production and composition

As indicated in Table 2, in this experiment, the supplementation of 10 g/d LPL in the diet of lactating dairy cows significantly enhanced milk yield (*P* < 0.05). In the analysis of milk components, both the milk fat percentage and milk protein percentage in the LPL group significantly increased compared to the control group (*P* < 0.05), while there were no significant influences on lactose, total solids, somatic cell count, and urea nitrogen content.

**Table 2:** Effects of LPL supplementation on milk yield and milk composition.

Items	CON	LPL	SEM	*P* value
Milk yield, kg	41.41	45.91	0.560	0.040
Fat, %	3.90	4.45	0.225	0.025
Protein, %	3.24	3.34	0.044	0.040
Lactose, %	5.09	5.11	0.052	0.638
TS, %	13.54	13.64	0.493	0.751
MUN, mg/dL	15.63	14.88	0.806	0.365
SCC, × 104/ml	25.57	24.63	7.104	0.896

Table 2 presents the effects of dietary LPL supplementation on lactation performance parameters in dairy cows. CON, control group; LPL, lysophosphatidylcholine group; TS, total solids; MUN, milk urea nitrogen; SCC.

### 3.2 Apparent nutrient digestibility

The results in Table 3 indicate that the addition of LPL to the diet of lactating dairy cows had no significant influence on the apparent nutrient digestibility of DMI, CP, NDF, and ADF (*P* > 0.05). In contrast to the CON group, the apparent digestibility ofEE in the LPL group increased significantly (*P* < 0.05).

**Table 3:** Effects of LPL on apparent nutrient digestibility in lactating cows.

Items	CON	LPL	SEM	*P* value
DM	75.22	79.86	1.27	0.26
CP	76.01	76.02	0.59	0.08
EE	59.37	69.56	2.12	0.04
NDF	36.46	34.39	1.02	0.34
ADF	23.64	26.42	1.26	0.33

Table 3 presents the effects of dietary LPL supplementation on apparent digestibility in dairy cows. CON, control group; LPL, lysophosphatidylcholine group; DM, dry matter; CP, crude protein; EE, ether extract; NDF, neutral detergent fiber ADF, acid detergent fiber. Data are presented as means ± SEM *P* < 0.05 was considered significant.

### 3.3 Blood biochemical parameters

As indicated in Table 4, compared with the control group, the supplementation ofLPL significantly increased blood glucose concentration by 36% (*P* = 0.024). Moreover, the urea nitrogen level in the LPL group was significantly lower than that in the control group (*P* < 0.05). However, no significant differences were observed in other serum biochemical parameters, including albumin and insulin, between the two groups. In addition, the LPL group exhibited a significantly higher concentration of NEFA (*P* = 0.001) and a significantly lower triglyceride content (*P* < 0.01), suggesting that LPL supplementation has a notable influence on lipid metabolism in dairy cows.

**Table 4:** Effects of LPL on serum biochemical indicators in lactating cows.

Items	CON	LPL	SEM	*P* value
TP, g/L	75.99	77.57	1.984	0.437
ALB, g/L	37.61	38.57	1.223	0.441
INS, uIU/L	16.77	17.09	0.719	0.661
GLU, mmol/L	2.67	3.64	0.380	0.024
AST, U/L	113.04	109.48	12.882	0.786
ALT, U/L	45.53	40.04	3.379	0.347
BUN, mg/dL	14.81	10.39	1.619	0.014
HDL-C, mmol/L	2.60	3.41	0.398	0.062
LDL-C, mmol/L	1.20	1.07	0.190	0.493
TC, mmol/L	8.03	7.89	0.716	0.847
TG, mmol/L	2.50	1.48	0.398	< 0.001
BHBA, mmol/L	0.63	0.54	0.088	0.085
NEFA, mmol/L	0.12	0.17	0.011	0.001

Table 4 presents the effects of dietary LPL supplementation on serum biochemical parameters in dairy cows. CON, control group; LPL, lysophosphatidylcholine group; TP, total protein; ALB, albumin; INS, insulin; G LU, glucose AST, aspartate aminotransferase; ALT, alanine aminotransferase; BUN, blood urea nitrogen; HDL-C, high-density Lipoprotein cholesterol; LDL-C low-density lipoprotein cholesterol; TC, total cholesterol; TG triglyceride; BH BA, β- hydroxybutyrate; NEFA, non-esterified fatty acids. Data are presented as means ± SEM. *P* < 0.05 was considered significant.

### 3.4 Rumen fermentation parameters

As depicted in Table 5, the content of MCP in the LPL group exhibited a marked increase in contrast to the CON group (*P* < 0.05), concurrently, the content of NH3-N in the rumen of the LPL group was significantly decreased (*P* < 0.05). We observed that the supplementation of 10 g /d LPL significantly increased both the concentration of propionic acid and its proportion in TVFA., yet had no impact on the final ratio of acetate to propionate. In comparison with the CON group, no significant disparities were observed in the contents of other volatile fatty acids in the rumen of the LPL group. There was no significant variance in TVFA, but a numerical increment was witnessed.

**Table 5:** Effects of LPL on rumen fermentation in lactating cows.

Items	CON	LPL	SEM	*P* value
pH	6.18	6.27	0.079	0.307
TVFA, mmol/L	115.83	123.03	5.809	0.243
Acetate, mmol/L	66.60	68.22	3.363	0.645
Propionate, mmol/L	26.35	32.03	1.665	0.014
Butyrate, mmol/L	11.59	12.11	0.511	0.330
A/P	2.16	2.44	0.161	0.121
**% of total VFA**				
Acetate	57.33	55.50	0.010	0.110
Propionate	22.50	26.00	0.006	< 0.001
Butyrate	10.17	9.67	0.007	0.511
MCP, mg/dL	94.31	102.04	2.778	0.011
NH3-N, mg/dL	22.07	18.33	1.501	0.023

### 3.5 Ruminal bacterial diversity and composition

The analysis presented in Figure 1 indicates that, in this experiment, no significant differences were observed in the Alpha Diversity indices of the rumen bacterial community (*P* > 0.05). The Shannon index values were relatively high across all sample groups, while the Simpson index values were relatively low, suggesting a relatively even distribution of species within the community. Additionally, the coverage for each sample exceeded 0.99. Therefore, it can be concluded that the rumen bacterial community exhibited high species richness and diversity, and the sequencing coverage was sufficiently comprehensive to accurately reflect the structural characteristics of the microbial community.

**Figure 1:**
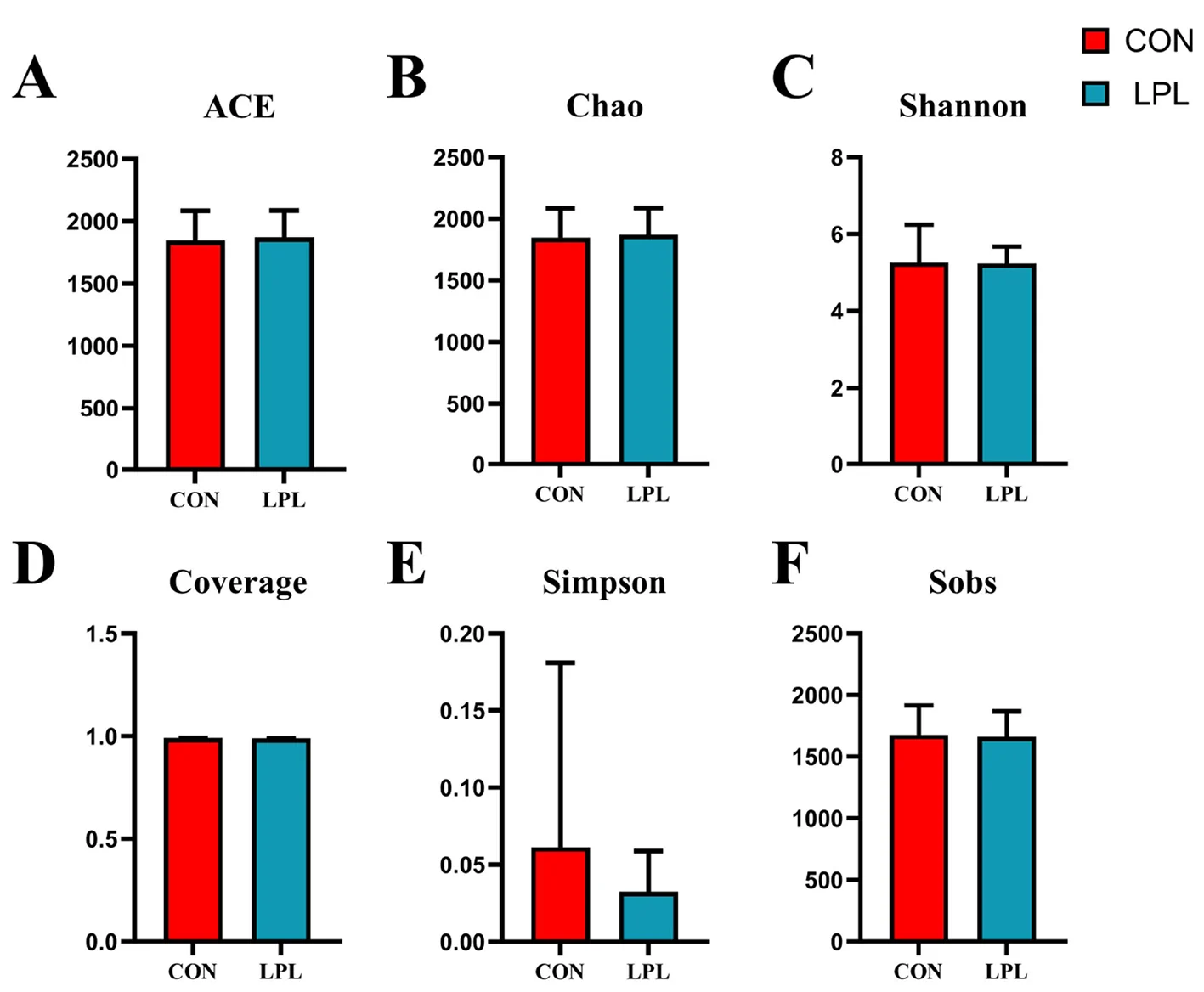
Graphical summary of the Alpha-Diversity index of rumen bacterial communities. The CON group was fed solely with the basal diet, whereas the LPL group received an additional supplementation of 10 g/d LPL on the basal diet. Data are expressed as mean ± SE, *n* = 6. ACE **(A)**, Chao **(B)**, Shannon **(C)**, Coverage **(D)**, Simpson **(E)**, Sobs **(F)**.

To visually illustrate the differences in microbial community structure, we employed PCoA analysis. As shown in Figure 2, the cluster separation between the CON group and the LPL group in both PCoA, 260 indicating that the differences in community composition between the two groups were not statistically significant. Further analysis revealed that the first two principal components, PC1 and PC2, explained 262 36.89% and 21.44% of the total variance, respectively. In conclusion, based on the PCoA analysis, it can be inferred that the CON group and the LPL group exhibit similar community compositions and species abundances, suggesting that the addition of LPL did not have a significant impact.

**Figure 2:**
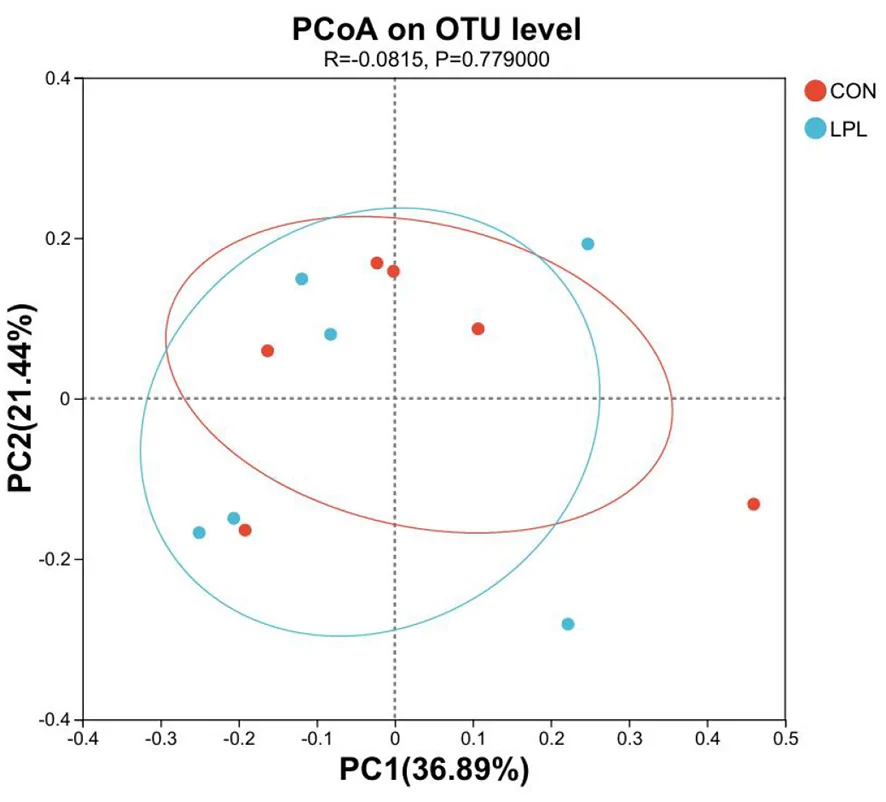
Principal coordinate analysis (PCOA) of rumen microbiota based on Bray-Curtis dissimilarity of OTU composition. *n* = 6.

As shown in Figure 3, based on the species annotation results, the top 10 most abundant phyla in each group were selected to construct relative abundance bar charts. In both the CON and LPL groups, *Firmicutes* and *Bacteroidetes* exhibited the highest abundances in rumen fluid, comprising 59.66% and 32.02% of the microbial community in the CON group, and 65.82% and 24.68% in the LPL group, respectively. These findings indicate that these two phyla are dominant within the rumen microbiota. At the genus level, 278 *Prevotella, Erysipelotrichaceae_ UCG-002, Succiniclasticum, NK4A214_group, Lachnospiraceae_NK3A20_group*, and *Christensenellaceae_R- 7_group* collectively accounted for over 45% of the microbial composition in both the CON and LPL groups, thus being classified as the dominant genera. We performed significance tests to evaluate the differences between the CON group and the LPL group at both the phylum and genus levels. The results indicated that, at the phylum level, the abundance of the *Patescibacteria* was significantly lower in the LPL group compared to the CON group (*P* < 0.0 5). At the genus level, the rumen microbiota of lactating dairy cows demonstrated high diversity. Notably, when compared with the control group, the relative abundances of *Candidatus_Saccharimonas, Novosphingobium*, and *UCG-009* were significantly reduced, whereas the relative abundance of *[Eubacterium]_ ventriosum_group* was significantly increased in the group supplemented with 10 g/ day of LPL (*P* < 0.05).

**Figure 3:**
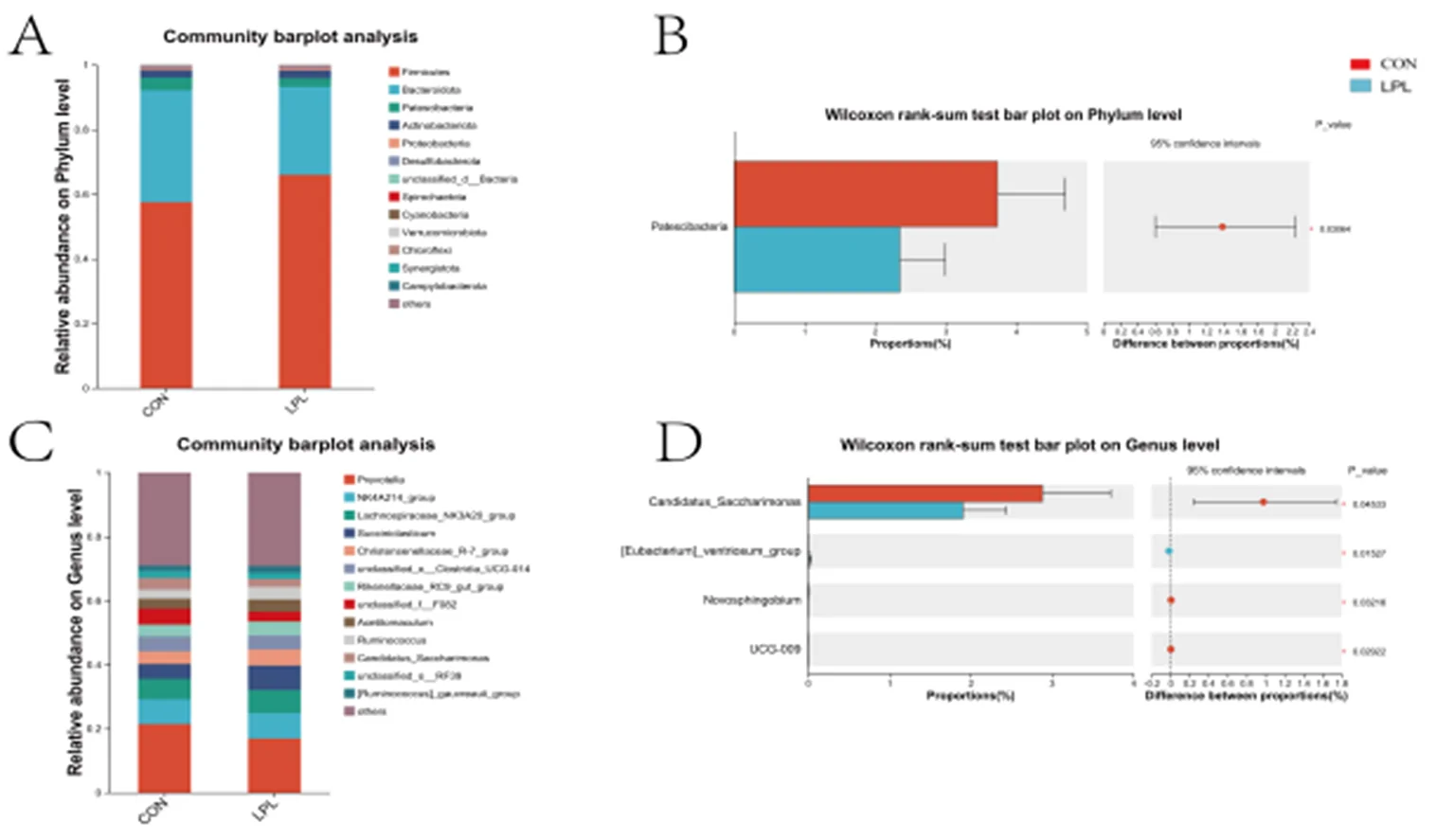
Species composition analysis revealed distinct microbial community structures at both the phylum and genus levels between the CON and LPL groups. Through species-level differential analysis, significant differences in microbial taxa at both the phylum and genus levels were identified between the CON and LPL groups. **(A)** Comparison of abundances of rumen microbiota at the phylum level in the CON and LPL groups; **(B)** Comparison of abundances of rumen microbiota at the phylum level in the CON and LPL groups; **(C)** Composition of abundances of rumen microbiota at the genus level in the CON and LPL groups. **(D)** Comparison of abundances of rumen microbiota at the genus level in the CON and LPL groups.

The differential bacterial analysis was conducted using an LDA score threshold of 2. Compared with the CON group, the abundance of *Rikenellaceae_family* and *Eubacterium_genus* was significantly higher in 297 the LPL group as shown in Figure 4. Conversely, the abundances of the *Patulibacteriota_phylum*, 298 *Novosphingobium_genus*, and *Butyricicoccus_family* were higher in the CON group relative to the LPL 299 group. Additionally, the abundance of *Gluconobacter* was consistently higher in the CON group than in the 300 LPL group across all taxonomic levels from order to genus.

**Figure 4:**
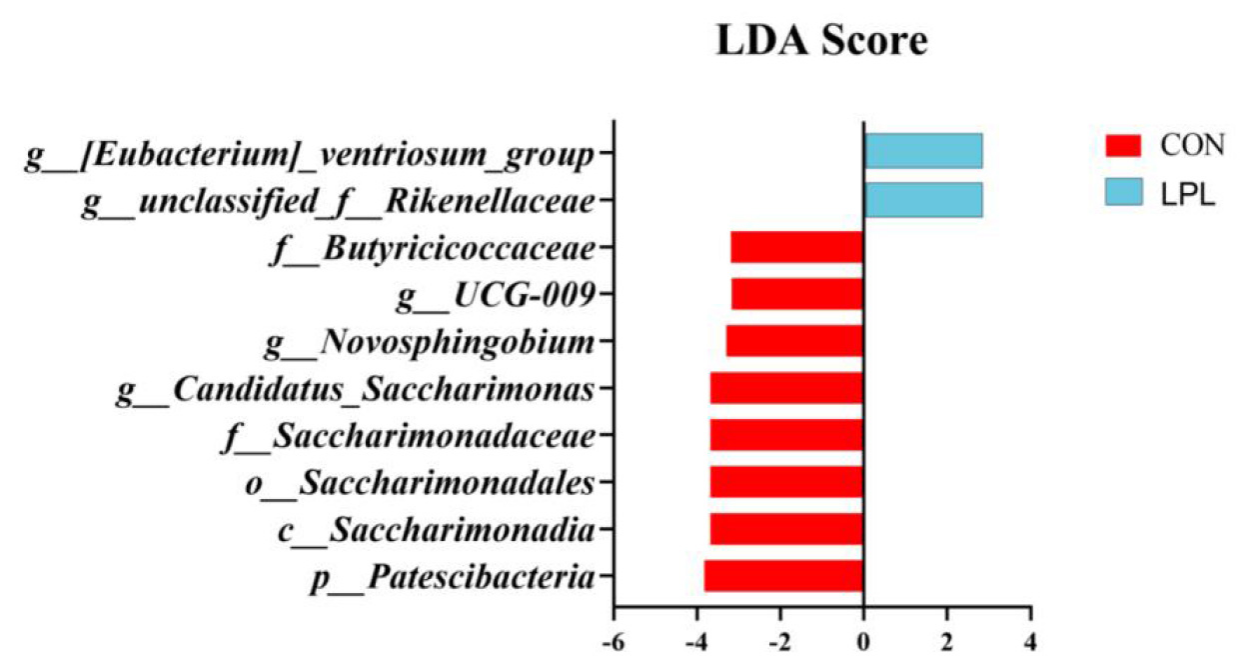
Using LEfSe differential analysis, we identified microorganisms in the rumen of the CON and LPL 292 groups that exhibited statistically significant differences (LDA score threshold > 2). The LDA score, derived from 293 LDA analysis (a form of linear regression), indicates that a higher LDA score corresponds to a greater influence of 294 species abundance on the observed differences.

We performed a correlation analysis between rumen fermentation parameters and specific serum biochemical indicators, with the results presented in Figure 5. The analysis revealed that the relative abundance of *Candidatus_Saccharimonas* was significantly negatively correlated with GLU (high statistical significance) and significantly positively correlated with TG. Furthermore, the relative abundance of *Erysipelotrichaceae_UCG-002* exhibited significant negative correlations with acetic acid and TVFA levels and an extremely significant negative correlation with propionic acid levels. Additionally, propionic acid content showed significant positive correlations with the relative abundances of *Christensenellaceae_R- 7_group, Rikenellaceae_RC9_gut_group, unclassified_f UCG-01 1, Butyrivibrio*.

**Figure 5:**
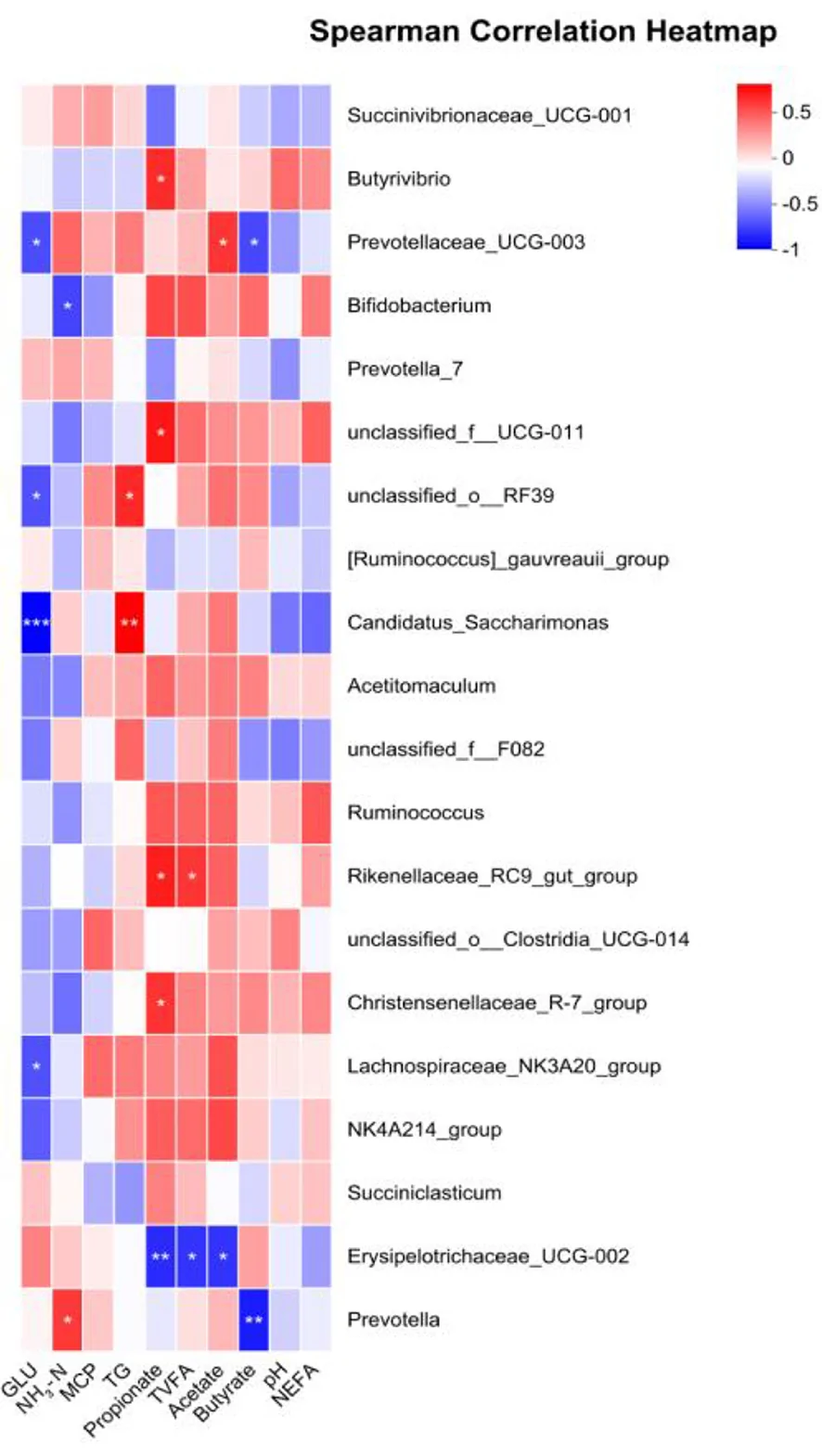
Correlation heatmap analysis was employed to investigate the impact of various environmental factors on the community composition of rumen microbial genera. Spearman correlations were calculated between rumen bacterial communities and phenotypic variables. The color scale represents the strength and direction of the correlation, with red indicating positive correlations (closer to 1) and blue indicating negative correlations (closer 307 to - 1). The intensity of the color reflects the magnitude of the correlation coefficient. *indicates 0.01 < *P* ≤ 0.05, **indicates *P* ≤ 0.01, ***indicates *P* ≤ 0.001 *n* = 6.

## 4 Discussion

Only a limited number of studies have evaluated LPL supplementation in dairy cows or other ruminants (7, 10, 19, 24). Therefore, the present findings were interpreted primarily in relation to evidence from dairy cows, lambs, sheep, and rumen-based studies, with particular consideration of ruminal lipid metabolism. Differences in LPL product composition, basal diet, dietary lipid concentration, physiological stage, initial production level, and supplementation duration were considered when comparing the results among studies.

In the present study, supplementation with 10 g/d LPL increased milk yield, milk fat percentage, and milk protein percentage. Rico et al. (24) reported that supplementation with 10 g/d lysolecithin increased milk fat synthesis under specificdietary conditions, whereas Lee et al. (7) and He et al. (19) observed limited orinconsistent effects of LPL on milk production in lactating cows. The variation among studies may be related to differences in LPL composition, basal diet, dietary lipid concentration, lactation stage, initial production level, and treatment duration. The production responses observed in the present study were accompanied by increased apparent EE digestibility. However, because intestinal lipid absorption and tissue lipid flux were not directly measured, these findings indicate an association rather than direct evidence of a specific emulsification mechanism.

LPL supplementation increased apparent EE digestibility without significantly affecting the apparent digestibility of DM, CP, NDF, or ADF. Lee et al. (7) similarly reported no significant changes in fiber digestibility following LPL supplementation in lactating dairy cows, while Huo et al. (10) found that dietary LPL affected nutrient digestion in lambs. These findings support the possibility that LPL can modify nutrient utilization in ruminants, although the response has not been consistent across studies. Rumen-based work indicates that surfactants can influence microbial enzyme activity (25), and dietary phospholipids have been reported to affect ruminal fermentation, nutrient digestion, and serum lipid profiles in sheep (26). In ruminants, dietary lipids undergo lipolysis and biohydrogenation in the rumen before the remaining lipid fractions reach the small intestine. Because ruminal lipolysis, biohydrogenation, intestinal lipid absorption, and lipase activity were not directly measured, the physiological basis of the increased EE digestibility remains to be determined.

Serum AST, ALT, TP, and ALB concentrations did not differ between treatments. The unchanged TP and ALB concentrations were consistent with theabsence of a treatment effect on apparent CP digestibility. Reis et al. (8) reportedhigher serum TP concentrations in calves receiving LPL in milk replacer, whereas He et al. (19) observed changes in selected biochemical indicators in mid-lactation dairy

LPL supplementation decreased ruminal NH3-N concentration and increased MCP concentration, propionate concentration, and the molar proportion of propionate, while pH, total VFA concentration, and the acetate-to-propionate ratio were not significantly affected. These findings indicate that selected aspects of rumen nitrogen and carbohydrate fermentation were altered. Lee et al. (7) reported only minor changes in rumen fermentation following LPL supplementation in lactating cows, whereas Jenkins (27) demonstrated that dietary fatty acid composition can modify ruminal fermentation patterns. Sontakke et al. (28) also reported nutrient-utilization and production responses to rice-bran lysophospholipids in crossbred cows.

LPL supplementation did not significantly affect alpha diversity or overall beta-diversity separation, indicating limited restructuring of the rumen bacterial community. Nevertheless, several taxon-specific differences were detected, including lower relative abundances of *Patescibacteria, Candidatus_Saccharimonas, Novosphingobium*, and *UCG-009*, and a higher relative abundance of the *[Eubacterium]_ventriosum_group*. Huo et al. (10) also observed changes in selected bacterial taxa in lambs receiving LPL, and an *in vitro* rumen study showed that LPL can affect the activity of selected lipolytic bacteria under a high-oil forage condition (29). These findings suggest that LPL may influence specific microbial populations without producing a broad shift in overall community diversity. However, changes in relative abundance alone do not demonstrate corresponding changes in microbial function or ruminal lipid metabolism.

The correlation analysis identified associations between selected bacterial genera and serum or rumen fermentation variables. *Erysipelotrichaceae_UCG-002* was negatively associated with propionate in the present study, which is partly consistent with observations in weaned lambs (30). *Prevotella* is widely involved in the utilization of non-cellulosic polysaccharides, starch, and nitrogenous substrates in the gastrointestinal tract of livestock (31), which may help explain its association with NH_3_-N and fermentation variables. Previous dairy cow and *in vitro* rumen studies have also reported associations between *Candidatus_Saccharimonas* and fermentation-related variables (32, 33).

## 5 Conclusion

Supplementation with 10 g/d LPL increased milk yield, milk fat percentage, milk protein percentage, and apparent EE digestibility in lactating dairy cows. LPL also altered selected serum biochemical and rumen fermentation indices, while overall rumen bacterial diversity and community structure were only modestly affected. The present results suggest that the production response was associated with improved fat utilization and changes in systemic metabolism; however, the underlying physiological mechanisms remain to be verified through direct measurements of ruminal lipid metabolism, intestinal lipid absorption, and tissue lipid flux.

## Data Availability

The raw sequencing reads were deposited into the NCBI Sequence Read Archive (SRA) database (Accession Number: SRP1290675), PRJNA1290675: https://www.ncbi.nlm.nih.gov/sra/?term=PRJNA1290675. The datasets used and/or analyzed during the current study are available from the corresponding author on reasonable request.
